# Research capacity-building for clinicians: understanding how the research facilitator role fosters clinicians’ engagement in the research process

**DOI:** 10.1186/s12961-022-00849-8

**Published:** 2022-04-27

**Authors:** Tracy Flenady, Trudy Dwyer, Julie Kahl, Agnieszka Sobolewska, Kerry Reid-Searl, Tania Signal

**Affiliations:** 1 School of Nursing & Midwifery, Central Queensland University, Building 18, Bruce Highway, Rockhampton, 4702 Australia; 2 Central Queensland Hospital and Health Services, Canning Street, Rockhampton, 4701 Australia; 3 School of Health, Medical & Applied Sciences, Central Queensland University, Building 6, Bruce Highway, Rockhampton, 4701 Australia

**Keywords:** Research capacity-building, Novice researcher, Research facilitator, Clinician researcher

## Abstract

**Background:**

There is evidence reporting more positive outcomes from research capacity-building (RCB) programmes that include a research facilitator role. Further, it has been suggested that research facilitator roles can be a useful strategy in building the research capacity of healthcare clinicians. However, until now, little attention has been applied to identifying the characteristics of the research facilitator role and how this role contributes to clinicians’ engagement with the research process. The aim of this present study is to explore the characteristics required of the research facilitator role in the educational workshop phase of an RCB programme.

**Methods:**

This qualitative study employed an inductive approach and utilized face-to-face interviews to gather data from a purposely selected cohort. Professionally transcribed responses were thematically analysed.

**Results:**

The role of the research facilitator emerged as comprising two main themes: (1) facilitating the research process and (2) engaging expert clinicians as novice researchers. Pragmatically, analysis of data led to the development of a table outlining the responsibilities, skills and attributes related to each theme. Conceptually, theme 1 encapsulates the research facilitators’ skills and experience and their role as knowledge brokers and cocreators of knowledge. Theme 2 provides insight into the clinician-centric approach the research facilitators utilized to build and foster relationships and support the clinicians through their research journey.

**Conclusion:**

This study reports on the characteristics of the research facilitator role in one phase of an RCB programme in one regional health service district in Australia and explains how the role fosters clinicians’ engagement with the research process. Findings from this study will inform the development of future RCB programmes, which is important considering that clinicians’ increased engagement with the research process is vital for developing a sound evidence base to support decision-making in practice and leads to higher levels of skills and greater ability to perform useful research.

## Background

Despite advances and innovation in healthcare and healthcare research, a gap between research and practice remains, with evidence to suggest it takes an average of 17 years for clinical research evidence to reach clinical practice [1–3]. Considering that only 60% of treatments provided by clinicians are evidence-based, research capacity-building (RCB) of clinicians is a priority if we are to narrow the research–practice gap [4]. RCB is defined as a “process of individual and institutional development which leads to higher levels of skills and greater ability to perform useful research” [5]. Capacity-building of clinicians can range from gaining an appreciation of the value of research and keeping up to date with contemporary research, to applying findings in clinical decision-making processes [6]. Additionally, capacity-building can progress to knowledge creation and conducting clinical research that has the potential to enhance the quality of patient care [7] and, ultimately, embed discovered knowledge into practice [8]. The relationship between knowledge discovery and implementation is iterative and embedded in healthcare practice [8, 9].

Healthcare is not only the consequence of research but is also the setting for research, in close relation to, and iterating between, healthcare and health research [9]. At the coalface, clinicians have the most practical understanding of issues important to practice, and exposure to research that addresses these problems results in greater questioning of the literature and current practices [10]. Despite clinicians being well positioned to be active in conducting research and not simply the recipients and potential users of research, a lack of research expertise is a significant barrier for clinicians undertaking research [11, 12]. Increasingly, health services are engaging research academics to facilitate the RCB of clinicians [13–15].

Implemented hospital-based RCB initiatives of clinicians typically involve partnerships between universities and hospitals [13, 16]. These may include the provision of educational research support, small research groups and academic research facilitators who act as mentors providing support and advice to design and carry out research projects. Such programmes contribute to the development of a positive research environment, directly affecting patient care [14, 17]. Facilitation has been popularized in healthcare as an innovation to bridge the chasm between knowledge generation and translation [18–20]. As a “guided interactional process” [18] and “dynamic interplay between multiple individuals” [19], facilitation is relationship-based and involves interactions between research “producers” and “users” [21]. Facilitation has evolved from a concept in the education and counselling literature to an implementation intervention in the healthcare and knowledge translation literature [20]. Researchers describe facilitation as both a role (the facilitator) and a process [18, 19]. Healthcare literature describes the term facilitator as an action, skill or contextual variable rather than an individual [19]. Roles such as the research facilitator can be a useful strategy in building the research capacity of healthcare clinicians [22–24]. However, there are gaps in understanding the potential of facilitation more broadly [18], and how it can be particularly applied to build research capacity of clinicians. While these examples offer a helicopter view of the usefulness of the research facilitator in building the research capacity of clinicians, the attributes of successful research facilitators are not well described in the current literature [19]. Our study sets out to examine the contribution of the research facilitator role in one RCB programme and to identify the characteristics of the research facilitator role to understand how this role contributes to clinicians’ engagement with the research process.

The RCB programme specific to this study, referred to as the Research Ready Grant Program (RRGP), was established in 2018 and can best be described as a multifaceted and multidisciplinary RCB programme developed to facilitate collaborative multidisciplinary practice–research partnerships. The RRGP consists of self-selected multidisciplinary (nursing, medical and allied health) teams of health professionals and multiple organizations (two universities and a hospital health service) working together to increase research capacity at the individual, team and organizational level. The programme is jointly funded by the health facility and one of the two partner universities (the other university provides in-kind support) and is delivered annually across a health service district in regional Australia.

The RRGP incorporates principles for successful capacity-building as presented by Cooke [6]. Multidisciplinary professionals from within the organizations work together to conceive a research idea that meets the organization’s research strategic plan. The organizational-level partners come together to provide the research mentor for the teams and form the RRGP committee to oversee the implementation of the programme. The RRGP engages research facilitators as a core component of the RCB programme.

The RRGP consists of two phases. Phase one comprises an 8-week series of educational workshops where multidisciplinary teams work together, and with the support of a research facilitator, turn a clinical problem into a researchable question and then develop a research proposal around that topic. Phase two involves a competitive, peer-reviewed, grant submission process whereupon teams from phase one submit their developed research proposals and vie for funding to operationalize their research proposal. As research supervision and mentorship are intrinsic to the successful completion of research projects [25], successfully funded teams are provided with mentor support for the duration of their project (phase 2). This study examines the experiences and perspectives of the research facilitators attached to the RRGP over a 2-year time frame to gain insight into how an embedded research facilitator role within an RCB programme facilitates clinician capacity and engagement in the research process. Of note, the component of the RCB programme reported on in this study is only concerned with phase one of the programme (the 8-week series of educational workshops).

## Methods

### Aim

This study aimed to explore the characteristics of an embedded research facilitator role in an RCB programme to develop an understanding of how this role contributes to clinicians’ engagement with the research process.

### Study design

This qualitative study employed an inductive approach and utilized semi-structured interviews to gather data from a purposely selected cohort. We conducted interviews with the aim of looking for patterns in the collected data and then generalizing results about any relationships between variables we identified. Thus, transcripts of all responses were thematically analysed.

### Participants and setting

To be eligible, participants had to have fulfilled the role of a research facilitator in the nominated RCB programme. Recruitment for research facilitators occurred over the three collaborating organizations (one hospital, two universities), with facilitators required to meet eligibility criteria to perform the role. There were nine expressions of interest from PhD-qualified academics to act in the research facilitator role, and as all nine met the criteria, all were accepted into the RCB programme. All nine (five women, four men) were interviewed, representing all the research facilitators participating in the RCB programme across 2 years. In other words, this sample size represents the total number of people who met the eligibility criteria for participation in this research study. The facilitators were known to the research team due to their research-active status within the universities, but there were no instances where members of the research team line-managed the facilitators.

### Data collection

Semi-structured interviews were chosen, as this option allows for the interviewer to pre-prepare questions designed to keep the interviewee on topic, while offering the flexibility to have a less formal conversation with participants where new ideas and topics may arise that can then be further explored [26]. This methodology encourages in-depth exploration of the phenomenon [27]. The interview guide and questions were pilot-tested. The fourth author conducted all interviews. Aside from the email communication to organize the interviews, she had no established relationship with the participants. The author at that time was working as a research assistant, and the participants were made aware of her role. The interviews were conducted via Zoom during business hours, with the option of participation through a video conference or audio only. Eight interviews were conducted as video conferences, and one as a teleconference. All interviews, which were conducted between late October and early December 2019, were recorded and later transcribed by a professional transcription service. The interviewer kept a reflective field journal during the data collection period. All interviews were limited to less than 60 minutes in duration, and during each interview, the following interview questions were asked:

- How does the research facilitator role contribute to building research capacity among clinicians?
- What attributes and skills does the research facilitator role require and why?
- Does the research facilitator role contribute to participants engaging with research? If so, how and why?

### Data analysis

The transcripts were returned to participants for comments. No changes were requested. Utilizing Braun and Clarke’s [28] thematic analysis methods, each transcript was manually coded by four members of the research team, with text extracts copied and pasted under inductively developed relevant coding categories into a Word document created for data analysis purposes. Data were then organized into categories to search for themes or patterns. The generated themes were then reviewed in a twofold process. Firstly, the research team examined the themes at the level of the coded data extracts. Secondly, the team reviewed the themes in relation to the data set and considered whether the themes reflected the meanings evident in the data set, all the while considering the themes in relation to each other. When consensus was achieved on the final definitions of themes, the resulting themes were named. The research team observed that the themes were being repeated across interview transcripts, which suggests having reached a level of completeness and thematic saturation [29]. The results of this thematic analysis were written up and shared with the participants as presented in the following section.

## Results

Following data analysis, two main themes emerged—*facilitating the research process* and *engaging expert clinicians as novice researchers*—that explain how the research facilitator role facilitates the capacity of clinicians to engage with the research process. Theme 1 relates to the facilitators’ research skills, knowledge, experience and research expertise, and theme 2 explores the facilitators’ teaching and learning, mentoring, engagement and communication skills. We present summaries of the two themes here, along with their subthemes, and provide examples of the collected data that support the development of these themes. Further, we have developed ready reckoner style tables for each theme, which present the reader with the responsibilities, skills and attributes related to each theme.

### Theme 1: Facilitating the research process

This theme, *facilitating the research process*, is densified by the identified subthemes, research skills and experience, making sense of the research process, knowledge brokers, and cocreating a positive research culture. Together, the overarching theme and subthemes explain how the research facilitator role facilitates the capacity of clinicians to engage with the research process.

#### Research skills and experience

The research facilitators brought their research experience into their roles. Participant 3 provided an overview of the general research characteristics of the RRGP research facilitators as a group: “*[they have]…a good mix of highly experienced researchers, with many years of publication, working with health services, getting external funding, and so on, through to some early-career researchers, who are highly enthusiastic about the things that they do, and really want to drive research in the same way that they were probably mentored as novice researchers in their early days*”. At a minimum, the research facilitators are early-career researchers with a focus on health services.

Research facilitators spoke about having the theoretical research knowledge coupled with the practical experience of conducting research. Participant 4 listed the skills and experience required for the role: “*…experience in research, conducting a range of different research projects so that the research facilitators have had experience addressing issues that come up—or identifying the issues that might come up in research, to help the clinicians to be aware of that and minimize the risk of those things happening in their own research. To be aware of different methods, research methods out there, that they could draw on to answer their research questions*”. Participant 2 also named the practical skills of project management including understanding of “*budget, the timeline, how to allocate the human resources and how to achieve each milestone in a timely manner*”.

#### Making sense of the research process

The research facilitators saw their role as simplifying the research process. In participant 9’s words, the role entailed: “*…support[ing] the clinicians and to help them think about the research process. So to step them through the very basics of how do you develop a research question, all the way through to really nailing down the research design*”. Each week, research facilitators had conversations with the teams of clinicians about how the content presented at the 1-hour lectures preceding the workshops related to their particular research projects. Participant 2 described the process as involving “*[making] sense of concepts…. not using so many jargons*”. Participant 4 elaborated: “*…there were a lot of unique studies [with] very unique sorts of issues that hadn’t necessarily been covered in the lectures, so the research facilitators were there to help*”.

Research facilitators offered guidance with developing feasible and achievable research proposals. The guidance included, as participant 6 described, “*…[making] sure that they use an appropriate design method, and data collection and data analysis methods… ethics is very important*”. Research facilitators recalled a trend of clinicians entering the RRGP with a mindset of wanting “*to save the world*”. The implication for the research facilitators’ role was that the research ideas were often too large and complex for the scope of the RRGP. Participant 5 recalled: “*Very often I had to pull them back… my biggest challenge was convincing them that it was just really more of a pilot project and a small project that they were looking at*”. Similarly, for participant 3, facilitation entailed “*breaking it down into something that is feasible*”. Research facilitators’ narratives suggest that they did not give directives but offered guidance in helping clinicians to make informed choices around designing achievable projects. Participant 6 reflected: “*I tried to show them how long did it take me to be able to collect my data. That was like an experience that I shared, and they understood*”.

Understanding the clinical context for conducting the research projects was flagged as important by some. Participant 6 advised that with clinical research, “*there’s an extra barrier, because you’re always thinking about patients, you’re always thinking about their safety and… it’s another obligation that you have to follow*”. Participant 7 identified that the research facilitator’s role focus on “…*reinforcing the processes of research, rather than the actual details of how to conduct it within their particular hospital system*” was a limitation. Participant 7 argued that “*there needs to be somebody who could potentially assist in that clinical situation, or understanding clinical context… somebody who's within the health system or within the hospital system, to negotiate some of those questions that arise that are very context-specific to health and to the hospital*”. Participant 7 argued that this clinical guidance could be fundamental in particular for preparing successful project ethics applications to the hospital’s ethics committee.

#### Knowledge brokers

The role of a research facilitator emerges as that of a knowledge broker. Research facilitators, as research experts in specialized fields, openly acknowledged their limitations in advising on all research matters. Participant 5 stated that “*…there were times where I felt my expertise wasn't matched enough and I could see that they [clinicians] needed that help… If they asked me specific questions, I'd say, look, I'm not an expert in breast cancer, but as an overarching research project, this is how that would normally be handled*”. While they were able to provide general guidance, the research facilitators connected the clinicians with other researchers that had the relevant expertise when required. Participant 8 noted: “*…if I wasn't competent with a group, or if I didn't know what they were doing, then I would always get external support*”. Research facilitators spoke about connecting clinicians with other RRGP research facilitators around research design issues. Participant 9 elaborated: “…*my background is very much in quantitative research, and if someone is looking at doing something from a qualitative perspective, there are other research facilitators who are more experienced in those qualitative methodological approaches… I would link them up with one of the other research facilitators that had more experience than I did, to be able to guide that expertise in that qualitative space*”. At times, research facilitators provided links to resources. Participant 1 spoke about doing follow-up work in between the workshops: “…*I might even do a little bit more research myself. Then take that information to the workshop the following week and just give it. Or email out afterwards*”.

#### Cocreating a positive research culture

There was a shared recognition of the RRGP driving the research culture over the long term. As participant 3 expressed, “…*it’s not just about that one individual project, but it’s the bigger picture of the relationship between the health service and the university*”. Similarly, participant 7 observed: “*RRGP programme is adding to being able to enhance the amount of research that's coming out of the hospital … it can only be of benefit in improving clinical practice within the hospital but also more broadly*”. As the RRGP is nested within the organizational structures and aligns with the strategy to increase the capacity of the health service to conduct research, it is designed as a longer-term investment around the RCB of the clinicians.

The 8-week programme is thought to be foundational to creating research culture. Participant 8 reflected that if clinicians fully engaged and regularly attended the RRGP, “*…it (the skills development programme) boosts their awareness of research. It boosts their social interaction with other like-minded people that are doing the same thing. It boosts their knowledge of all the foundations of research*”. In participant 4’s experience, “*…the clinicians were able to identify immediate needs for research in the real world and to be able to use my skills to help… to draw up a plan to be able to address that need and do research to solve that problem*”. Participant 1 observed: “*I hear the language change in their voice when they start to talk about it [research]. I really think that once they understand how engaging with research impacts their professional role, and their ability to effect change, really, and how they can incorporate what they’ve researched into their everyday practice, I don’t think they can turn back*”. Participant 3 elaborated on the potential of a bottom-up effect in the diffusion of the research culture on the peer-to-peer health service level: “*…they [the clinicians] can then go off and go, okay, we learnt these skills in the previous project, how can we apply that to something new, that we might want to do, without being part of the project… They can then inspire… others that, yeah, you know what, research actually isn’t such a bad, nasty, scary thing to do, that others might at least be willing to join the programme and have a go at it*”.

The RRGP was a platform for knowledge exchange between health services and academia. Participant 4 expressed the value of seeing the fast progression of the real-world research process, in contrast to academic research which can be slow in terms of dissemination and achieving the translational impact. Participant 9 expressed reward from being “*a part of translating research to practice*”. Participant 6 reflected: “*…there were problems that I’ve never thought of. There were solutions that I’ve never thought of … because every discipline has their own viewpoints, there were discussions at the table [a lot of], I guess a lot of advantages in terms of me understanding what my future research, in terms of clinical research, can be*”. Participant 6’s comment mirrors the sentiment expressed by other research facilitators, not only about the professional value in learning about the current issues in healthcare, but also in being part of envisioning new approaches for investigating and solving these issues through research.

During the interviews, the research facilitators were asked about the skills and attributes they believe are important to the research facilitator role. Table 1 lists the responsibilities, skills and attributes that research facilitators identified. The responsibilities relate to the scope of the research facilitator’s role over an 8-week period or the duration of phase 1 of the RRGP in working with clinicians to help them develop research proposals. The identified skills relate to theoretical and practical understanding of research methods as well as designing and conducting research projects. Attributes relate to the acknowledgement among the research facilitators that it is unrealistic for an individual to have all the answers and that the role entails facilitating links with other research experts and appropriate resources.

**Table 1:** Responsibilities, skills and attributes for facilitating the research process

Responsibilities	Skills	Attributes
• Working with clinicians as novice researchers to help them develop research proposals • Simplifying the research process • Answering general questions about research • Discussions with clinicians about what they learned in the lecture meant for their study • Improving practice ideas and concepts into study • Breaking down projects into feasible ones • Providing support on how to formulate research questions, identify gaps, choose suitable study methods • Working with groups with different levels of research knowledge • Keeping participants on track • Research facilitators as resources: linking up with people, linking to appropriate resources	• Experience in research design and the fundamentals of conducting a research project • Qualitative or quantitative research design experience • Completing ethics, hospital ethics • Research project management required—budget, human resources, how to achieve each milestone in a timely manner • Can identify issues that come up with research • Understanding clinical research • Ethics applications • Grant applications	• Conduit—a link between—providing information to clinician • “I’m not a cancer expert, but…” • Understanding own limitations as to how the research facilitator can contribute to the group • Overcoming the gaps in personal areas of expertise when dealing with different research groups

### Theme 2: Engaging expert clinicians as novice researchers

Engaging expert clinicians as novice researchers emerged as a major theme in narratives about the skills and attributes that RRGP research facilitators require if they are to build research capacity of clinicians who are acknowledged discipline experts and, yet, novices when it comes to conducting research. This theme is further explained by the inclusion of the identified subthemes: working with clinicians as novice researchers, clinician-centred approach to facilitation, building and fostering relationships, and supporting clinicians through their research journey. Together, theme 2, along with its subthemes, further explains how the research facilitator role facilitates the capacity of clinicians to engage with the research process.

Table 2 shows the responsibilities, skills and attributes related to this theme that research facilitators identified. These relate to group work and clinician-focused facilitation methods which were purposefully applied to engage clinicians in the research process. In phase 1 of the programme, the research facilitators work with “groups” of research projects.

**Table 2:** Responsibilities, skills and attributes for engaging expert clinicians as novice researchers

Responsibilities	Skills	Attributes
• Assess the needs of each research team and the group as a whole • Manage multiple research groups • Assess understanding of the weekly topic and develop the topic • Check for misunderstandings • Facilitate conversation in a safe space • Make material relatable on a professional and personal level • Encourage reciprocity of learning between the different participants • Provide written examples, exercises, extra resources, homework • Work with research emotions of the clinicians • Listen to clinicians as to what they want to do rather than impose own bias • Keep the enthusiasm for research • Instil confidence • Get to know participants personally and how they react to certain questions and content to support them accordingly • Work with negative emotions and emotional reactions to research • Break down professional hierarchies if applicable	• Motivational interview to help clinicians clarify a clinical problem into a research question • Using the collective wisdom of the group • Being able to see deficits in learning and learning needs • Good understanding of adult learning concepts—content delivered in easily digestible ways • Outstanding communication skills • Active listening • Looking and observing • Clinician background of research facilitators can be useful • Allowing everyone to have a voice • Understanding communication cues • Developing rapport • Organizational skills to manage multiple projects, time management	• Open mind • Being adaptable—absorb information quickly • Being aware of own biases and ensuring that they don’t interfere (e.g. spending more time with groups with shared topic expertise) • Transparency and authenticity to meet the clinicians’ expectations • Enthusiastic approach • Being approachable

#### Working with clinicians as novice researchers

Working with clinicians as novice researchers involved working with their clinical expertise. While research facilitators indicated that the research skills among clinicians varied due to the absence of or limited research training, clinicians had the necessary clinical expertise. In participant 3’s words, the clinicians “*see these research-worthy things on a day-to-day basis… they participate in the research by really leading the design… I would lean on their knowledge and experience*”. The presence of a practice–research continuum would emerge from the research facilitators’ narratives. Participant 2 reflected that “*the clinicians have a lot of information … from their own experiences, but they don't know how to test this knowledge, how to test their assumptions*”. The research facilitators work with the complementary clinical and research roles. Participant 2 explained that “*there is sufficient knowledge base [that] … require[s]… to translate this knowledge to experiment in the research … Naturally we all are researchers. We always think rationally, we think logically*”. Participant 6 noted a difference between research and clinical work: “*clinicians always follow the guidelines and the rules … Those guidelines and processes are not always clear in research*”.

Clinicians and their projects bring strengths to the research–practice continuum. Participant 2 observed that “*most of them (RRGP research projects) are clinical studies. The participants are clinicians… so they know their projects*”. Participant 1 also noted that “*participants are already motivated to come on their own time …which means that they want to be there and work through the problems… The goal at the end of the 8 weeks is to have a fully developed research proposal, and all of the teams did that. So that shows that they engaged with the process properly and that the research facilitator aided in their understanding of the material*”.

#### Clinician-centred approach to facilitation

A clinician-centred approach to facilitation was evident throughout the narratives. Research facilitators spoke about their focus on understanding and meeting the needs of the clinicians. Participant 1 commented: “*It’s my job to really assess the needs of each of the teams individually as well as a whole… pick up early any misunderstandings that they’ve got… Then extrapolate more on areas of the topic that they are showing a poor understanding*”. Research facilitators spoke about using active listening and observational skills to guide their assessment. Participant 1 explained: “*I listen to the questions they ask and encourage them to speak amongst themselves… but I really listen to the questions, and how they’re interacting around the topic*”. Participant 5 further noted: “*…you can just see in their body language that they're struggling with a topic*”.

Research facilitators spoke about exercising awareness of their own research bias and minimizing it. Participant 4 gave an example from working with one clinician: “*I saw lots of interesting studies and well-controlled studies, but the questions that she [the clinician] was asking—was wanting to ask based on her experience—were just different to what I thought would be interesting*”. Participant 4 noted how important it was “*to listen to the clinicians themselves as to understand what they are really wanting to research and what questions they want to answer without putting on our own interests of a research role or our own area that we specialize in*”. Research facilitators used adjectives such as being open, honest, frank, transparent and authentic in their clinician-centred approach. For participant 3, being open involved using “*…a motivational interviewing style, I guess, to help them [clinicians] clarify what they see as research-worthy, and turn that into a research question …asking lots of seemingly naïve questions, about why that’s important, and what are the implications of that and what’s been done about that in the past, and how does that fit with the working group that they’re attached to, and so on, so that I can get a clearer picture of what it is they’re trying to achieve and why*”.

#### Building and fostering relationships

Building and fostering relationships between the research facilitators and the teams, and between team members, was part of working with the clinicians to help them achieve research goals. In participant 1’s words, “*relationships are crucial to the learning process*”. There is a sense that through the relationship, research facilitators gained awareness of the learning needs of the clinicians. Participant 5 reflected that “*…you get to know them personally quite well, so you get to know what they're going to be worried about and how they learn and react to certain questions*”. Participant 5 further indicated that these relationships would guide how she structured the social environment for learning: “*…often people felt a little bit uncomfortable talking in front of that big group… the tactic I used … was to get them then into their own individual groups and then I would rotate around. One of the other research facilitators gave more of a lecture style workshop, like a tutorial, to the whole group, but that wouldn't have worked for me because they [project teams] were very different [from each other]*”.

Relationships within and between teams were conducive to reciprocity of learning among the clinicians. Participant 8 described the typical composition of the RRGP research team: “*…people in different departments, or different disciplines, that are like-minded … and have the same goal, with the same research idea*”. Participant 6 emphasized that the multidisciplinary teamwork “*…caused some good debates … which helped them to understand there are different barriers, and they can find a way… to overcome that barrier and solve those problems*”. Participant 8 pointed to the usefulness of the question “*what would you do from a [discipline-specific] perspective?*” to elicit problem exploration among the different disciplines and to “*mould their multidisciplinary team project into a nice, collegial project that can benefit all professions*”. Participant 1 saw the group discussions between the teams as enhancing adult learning. She observed that “*…everybody’s hearing everybody else’s responses, and it’s growing their understanding of it as well, and covering off on questions that perhaps they might have had anyway, which gives them the opportunity then to think more and extend their understanding of it and ask another question*”.

The research facilitator’s role at times required, in participant 5’s words, “*build[ing] capacity between the different members of the group*”. Participant 3 elaborated: “*You have a group or an individual that was… dominant in some particular way, then the success of that large group would have relied on the skills of the research facilitator to acknowledge that expertise but ensure that expertise was usefully directed to the group as a whole*”. For participant 5, facilitation involved shifting the “*imbalance of power*” in a team where the interprofessional hierarchy dynamics were playing out. Participant 5 spoke about “*facilitating the conversation in a safe space… ensuring that there was equal space and time for each of the participants*”. She observed that this process encouraged “*listening to different people’s opinion*” and facilitated a *“natural evolution*” within the first part of the RRGP workshop phase. Participant 5 observed that the particular group “*became much more open*” and “*a certain group of people were allowed to voice their opinion more strongly and have some say in the direction of their project*”.

#### Supporting clinicians through their research journey

“*Keeping the participants on track*” was the phrase used by research facilitators to convey the practical focus of their work. In participant 5’s experience, the teams “*would go away each week and they'd come back with completely different ideas. A lot of the beginning parts of the session was getting them back to being… on the same page*”. Over the course of the RRGP skills development programme, the research teams would also make different degrees of progress towards their research project proposals. Participant 7 reflected: “*Each of those groups are so different in what they're doing, it's very difficult to provide just a single comment about something to address everybody's needs*”. Research facilitators would allocate their time accordingly among their research groups. Participant 8 indicated that “*dividing your time relatively evenly between the groups is sometimes a little bit tricky when you've got some groups that you're very experienced in and so you can offer a lot more to that group because of your background experience*”.

Responding to the needs of the clinicians’ research groups was not overly complex. Research facilitators drew on their academic experience related to their organizational skills and adaptability. As participant 8 explained, “*we do that [manage multiple projects] with different PhD students, with different teaching into different units… often balancing our time between different things and different projects, and writing our own research, writing our own grants*”. Similarly, participant 5 identified the usefulness of her “*academic experience working with other researchers, doing some lecturing, being thrown in the deep end… to be adaptable and be able to absorb information quickly and then tilt it to how you need*”.

Working with a broad range of clinicians’ emotions at different stages of the RRGP skills development phase emerged from the narratives. Research facilitators commonly spoke about clinicians’ enthusiasm and saw it as an energizing force. In participant 1’s words, the group’s enthusiasm was “*contagious and exciting*”. However, frustration, fear, being overwhelmed, anger and disappointment had also surfaced. Participant 6 spoke about debunking the misconceptions about research at the beginning stage as part of the preparation. Participant 1 reported that “*they think it is very easy to put in the application, but when they see how [thorough] an ethics application process they, most of the time, get very scared of it. That can be a big barrier for our clinicians. Helping them understand that and helping them preparing for that in advance*”. Research facilitators also spoke about the emotional tensions among participants at the later stage of the skills development programme. Participant 5 noted the competition among the teams in their efforts to obtain funding for their proposals. In response, the research facilitator “*look[ed] at the cost with them and their budget, and … encouraged them to … keep going with it even if they don't get the funding*”. Participant 9 recalled an example of a “*very, very passionate clinician that ended up just throwing it in because it was just too hard*”. This was the most extreme example, highlighting that not all of the participants would go on to the next stage of the RRGP in terms of conducting the research studies. Dealing with unsuccessful proposals was on occasion the downside for the research facilitator and the clinicians.

Overall, to use participant 9’s words, the research facilitators regarded the RRGP as being “*a positive learning experience*”. Participant 1 emphasized the importance of sharing real-life examples during the workshops to make them relatable and engaging. She elaborated: “*Ethics week can be a really boring topic to cover, so I try to make it interesting by giving them real-life examples of situations that I’ve had with ethics applications, and make it more human, more emotional. These are things that happened to me, and usually it was because there were people involved with personalities, and this is something that I’ve learned*”. Research facilitators with clinical experience similar to some of the clinicians suggested its usefulness in terms of having shared reference points. For example, participant 2 remarked: “*When I talk about the clinical information, as I am a clinician also, the clinicians easily can get it and I’m hoping that—I’m sharing that with you now so that you can learn from it*”.

A sense of mutual commitment and investment between the clinicians and research facilitators emerged from the narratives. Research facilitators spoke about the time-consuming nature of RRGP participation for the clinicians in a voluntary, unpaid capacity. Reflecting on the clinician’s estimated 5- to 10-hour weekly commitment to the programme, participant 3 remarked: “*I also get the feeling they do it as a sense of commitment to us research facilitators, that, you know, we are giving up loads of our time, that they should do the same, to get the best out of the project*”. Research facilitators spoke about investing their time in supporting clinicians outside the realm of the workshop hours. They spoke about their commitment to the programme in terms of their commitment and fulfilment with mentoring novice researchers in conducting research, as well as the real-world significance of the implications of the RRGP research projects. Participant 9 summed up the reciprocity of investment: “*without the research facilitators, the RRGP wouldn’t exist, and without that programme, there’s no platform for researchers to engage in research activities like those that are presented within the RRGP*”.

## Discussion

Despite the recognition of the value of RCB, the available research about the effectiveness of efforts for building research capacity in healthcare organizations is limited in scope [30–32]. Historically, RCB interventions have included provision of support to individuals rather than groups, through fellowships, training schemes and bursaries [6, 22]. The effectiveness of RCB interventions has focused on identifying improvements in either the research skills of the individuals or the research outputs including journal publications, successful grant applications and conference presentations—outputs that are difficult for novice researchers to achieve [22, 33]. Our study highlights the importance of the research facilitator role in terms of facilitating the research process and engaging expert clinicians as novice researchers. The RRGP research facilitator identified that they achieved this by tapping into their theoretical research knowledge and drawing upon their practical experience of conducting research. The research facilitators identified their role as knowledge brokers who worked with the clinicians as novice researchers to simplify the research process and support them in turning a clinical problem into a feasible research question and then keeping them on track while they developed their research proposal. Attributes of successful research facilitators are not well described in the current literature [19]. Much of the facilitation literature focuses on how facilitation is conceptually useful for the “doing” phase of integrating research findings into the organizational settings [18]. However, simply creating knowledge in isolation does not drive clinical outcomes [19]. Until now, little attention has been applied to developing an understanding of the characteristics of the research facilitator role and how this role contributes to clinicians’ engagement in the research process. Our study reports on the characteristics of the research facilitator role in an RCB model and explains how the role fosters clinicians’ engagement with the research process.

The RRGP facilitators’ accounts suggest the potential of the role to develop research capacity in professional development context, outside of the traditional university environment. The second theme of engaging expert clinicians as novice researchers unpacks the facilitator’s role in helping the clinicians bridge the practice and research divide. As the subthemes reveal, the facilitator leads a research process which is clinician-centred, encompasses support and guidance and fosters relationships. Consistent with the constructivist approach, the facilitator helps the clinician learners to reconceptualize problems and solutions in a learner-centred environment [34, 35]. Clinical practice issues are turned into research projects. In line with constructivism, the learning occurs through the creation of meaning from experience, rather than the traditional knowledge transfer from an educator to the student [34]. The facilitator guides the clinicians in looking at familiar clinical problems from a new perspective. The meaning-making is a social process, situated within the respective multidisciplinary group dynamics of the participating clinicians. The multidisciplinary groups work together through the research process. As has been uncovered, research facilitation at times means building capacity between the different members of the group.

The facilitator leads in reconceptualizing the familiar contexts of clinical practice as sites for conducting research for the clinicians. The work contexts are experienced in a new way. This is consistent with the constructivist position, which assumes that knowledge transfer can be facilitated by involvement in authentic tasks which are anchored in meaningful contexts [34]. In this instance, the facilitator assists clinicians in their work environments to engage in research that is close to their practice. Ertmer and Newby [34] observed that the authenticity of the experience becomes critical to the learning process, and the context forms an inexorable link with the knowledge embedded in it.

The term “knowledge broker” has been applied to various contexts in healthcare settings [36], but fundamentally, the term is most often understood to mean a person who connects science and society. Whilst numerous publications provide information about the desired characteristics of a knowledge broker when translating evidence to practice [37–40], currently, little is known about the qualities required of knowledge brokers in terms of building clinicians’ research capacity. The RRGP research facilitators provided this study with rich and detailed data regarding the skills and attributes they believe are necessary when facilitating clinicians’ engagement with the research process, presented in detail in Table 1. The RRGP research facilitators explained the importance of providing advice on research matters in terms the clinicians would understand, but also acknowledging their limitations when need be.

There is reported evidence of RCB programmes in which health practitioners are acknowledged as novice researchers, which goes on to mention the inclusion of research facilitators or mentors throughout the RCB programme [13, 14, 22, 41–43]. These studies cite various positive consequences of including a research facilitator/mentor role in the RCB programmes; for example, the novice researchers felt greater support and valued the partnership [13], and there was evidence of an improved research culture [22] and increased publication outputs attributed to the inclusion of the role [41]. Whilst much has been written about the outcomes, there is limited information about the characteristics of the research facilitator when they were engaging with the expert clinicians as novice researchers. Results from our study will be useful when developing the position description for a research facilitator for future RCBs. Considering the amount of evidence highlighting the benefits of including a research facilitator in RCB programmes, it is important to understand the responsibilities, skills and attributes required to fulfil the role.

### Limitations of the study

Facilitators expressed a desire to continue to be involved in the programme, which may introduce a bias in how they self-reported and perceived the facilitator role. At the time of the interviews, most continued to be involved in the second phase of the programme as mentors for funded research teams. Perhaps because the facilitators had already dedicated much time and a great deal of effort over an extended period of time, they saw their participation as a long-term investment in terms of developing industry–research partnerships. Therefore, their appraisal of the facilitator role in the 8-week course may have had a more long-term focus. Another perceived limitation could be the low number of interviews conducted (nine). However, the RCB involved nine research facilitators over the 2-year time frame under investigation; all nine were invited to participate, and all nine accepted. Therefore, the number of interviews was directed by the number of available participants.

## Conclusion

There is evidence reporting more positive outcomes from RCB programmes that include a research facilitator role, and it has also been suggested that roles such as the research facilitator can be a useful strategy in building the research capacity of healthcare clinicians. However, until now, little attention has been applied to identifying the characteristics of the research facilitator role and how this role contributes to clinicians’ engagement with the research process.

This study reports on the characteristics of the research facilitator role in an RCB model in one regional hospital health service in Australia and explains how the role fosters clinicians’ engagement with the research process. We found that the research facilitators’ contribution can be characterized under two main themes: facilitating the research process and engaging expert clinicians as novice researchers. Analysis of data facilitated the identification of a table of responsibilities, skills and attributes required to fulfil the research facilitator role. This information will inform the development of future RCB programmes and is important when you consider that clinicians’ increased engagement with the research process is important for developing a sound evidence base to support decision-making in practice and leads to higher levels of skills and greater ability to perform useful research.

## Data Availability

The data sets generated and/or analysed during the current study are not publicly available due to the material being related (in some instances) to patient admissions. However, de-identified data may be available from the corresponding author on reasonable request.
